# Analysis of microRNAs Expression Profiles in Madin-Darby Bovine Kidney Cells Infected With Caprine Parainfluenza Virus Type 3

**DOI:** 10.3389/fcimb.2018.00093

**Published:** 2018-03-29

**Authors:** Jizong Li, Li Mao, Wenliang Li, Fei Hao, Chunyan Zhong, Xing Zhu, Xinqin Ji, Leilei Yang, Wenwen Zhang, Maojun Liu, Jieyuan Jiang

**Affiliations:** ^1^Institute of Veterinary Medicine, Jiangsu Academy of Agricultural Sciences, Nanjing, China; ^2^Key Laboratory of Veterinary Biological Engineering and Technology, Ministry of Agriculture, Nanjing, China; ^3^School of Pharmacy, Linyi University, Linyi, China; ^4^College of Animal Science, Guizhou University, Guiyang, China

**Keywords:** madin-darby bovine kidney cell line, caprine parainfluenza virus type 3, high-throughput sequencing, microRNAs, host-pathogen interactions

## Abstract

Caprine parainfluenza virus type 3 (CPIV3) is a newly emerging pathogenic respiratory agent infecting both young and adult goats, and it was identified in eastern China in 2013. Cellular microRNAs (miRNAs) have been reported to be important modulators of the intricate virus-host interactions. In order to elucidate the role of miRNAs in madin-darby bovine kidney (MDBK) cells during CPIV3 infection. In this study, we performed high-throughput sequencing technology to analyze small RNA libraries in CPIV3-infected and mock-infected MDBK cells. The results showed that a total of 249 known and 152 novel candidate miRNAs were differentially expressed in MDBK cells after CPIV3 infection, and 22,981 and 22,572 target genes were predicted, respectively. In addition, RT-qPCR assay was used to further confirm the expression patterns of 13 of these differentially expressed miRNAs and their mRNA targets. Functional annotation analysis showed these up- and downregulated target genes were mainly involved in MAPK signaling pathway, Jak-STAT signaling pathway, Toll-like receptor signaling pathway, p53 signaling pathway, focal adhesion, NF-kappa B signaling pathway, and apoptosis, et al. To our knowledge, this is the first report of the comparative expression of miRNAs in MDBK cells after CPIV3 infection. Our finding provides information concerning miRNAs expression profile in response to CPIV3 infection, and offers clues for identifying potential candidates for antiviral therapies against CPIV3.

## Introduction

Parainfluenza virus type 3 (PIV3) is an enveloped, single-stranded negative sense RNA virus within the *Respirovirus* genus of the *Paramyxiviridae* family, which is one of the most common causative agents of respiratory infection in different host species (Maidana et al., 2012). Three *Respirovirus* genus species are known to infect human (human parainfluenza virus type 1 and 3, HPIV1, and HPIV3), bovine (bovine parainfluenza virus type 3, BPIV3) and mouse (Sendai virus) (Ellis, 2010). The clinical features of bovine infection with BPIV3 varies considerably, ranging from subclinical infection to severe respiratory disease. In most of the cases where BPIV3 is implicated in disease, mild clinical signs characterized by coughing, pyrexia, nasal, and ocular discharge are observed (Horwood et al., 2008). In some circumstances, when animals undergo high stress, such as during temperature, humidity, stocking density, transportation, and other factors, BPIV3 infection contribute to tissue lesion and immunosuppression, leading to severe bronchopneumonia from secondary bacterial infections (Haanes et al., 1997; Wen et al., 2012). The resulting infection is part of the bovine respiratory disease complex (BRDC) and it is considered as the most important illness associated with feedlot cattle in China (Shi et al., 2014), USA (Snowder et al., 2006), and possibly worldwide (Maidana et al., 2012).

Since August 2013, a novel PIV3 (caprine PIV3, CPIV3) was detected from goat herds with respiratory illness around China through RT-PCR assay and nucleotide sequence analysis (Li et al., 2014; Li J. et al., 2016; Yang et al., 2016), the diseased animals associated with coughing, nasal discharge and dyspnea. Subsequently, the following study revealed the detailed pathogenicity characterization and horizontal transmission ability of the CPIV3 strain JS2013 in conventional goats (Li W. et al., 2016). However, most research is mainly concentrated on the epidemiology, phylogeny, transmission, pathogenicity, and immune response (Li J. et al., 2016; Li W. et al., 2016), with few studies focusing on its pathogenic mechanism. Therefore, this study focusing on non-coding RNAs during CPIV3 infection and it would provide new insight into understanding the molecular mechanisms of virus pathogenesis.

MicroRNAs (miRNAs), produced by both hosts and viruses, are an abundant class of short, endogenous, non-coding RNA molecules (19–24 nt in length). Mature miRNAs are incorporated into the RNA-induced silencing complex (RISC), leading to either mRNA degradation or translational repression by binding semi-complementarity to target mRNAs, and usually located in the 3′ untranslated regions (3′ UTR) (Bartel, 2004, 2009). These miRNAs are involved in a wide range of biological processes such as developmental timing, immunity, tumorigenesis, apoptosis, signal transduction and cell proliferation (Carrington and Ambros, 2003; Ambros, 2004; Schickel et al., 2008; Inui et al., 2010). Therefore, many studies have focused on the roles of miRNAs as modulators of host cell-virus interaction networks (Scaria et al., 2006; Grassmann and Jeang, 2008). Since virus infection can trigger changes of cellular miRNAs profile and these miRNAs can highly influence viral propagation and pathogenesis (Zhang et al., 2015). In recent years, researchers have demonstrated the impact of viral infections on the cellular miRNA profile; such as hepatitis C virus (Norman and Sarnow, 2010), Japanese encephalitis virus (Zhang et al., 2015), dengue fever virus (DENV) (Liu et al., 2015), and bluetongue virus (Xing et al., 2016). *In silico* analysis of the overall miRNAs and their targets suggest that they may be involved in viral latency.

The madin-darby bovine kidney (MDBK) cells was the ideal cell line for isolating and identifying a large number of virus, such as bluetongue virus (BTV) (Wang J. et al., 2017), orf virus (ORFV) (Zhao et al., 2017), hobi-like pestivirus (Mao et al., 2012), border disease virus (BDV) (Mao et al., 2015), bovine viral diarrhea virus 1 (BVDV-1) (Ni et al., 2015), and bovine herpesvirus 1 (BoHV-1) (Saha et al., 2013), moreover, the pathogenic mechanism of some of these virus were further evaluated in this host cell line (Zhao et al., 2017; Zhu et al., 2017; Shi et al., 2018). Recently, a great number of miRNAs have been reported to show altered profiles during infection and regulate the host immune responses (Fu et al., 2017; Lai et al., 2017). After the MDBK cells were infected with bovine viral diarrhea virus (BVDV), bta-miR-29b was significantly upregulated, and overexpression of bta-miR-29b led to the attenuation of BVDV infection-related autophagy, moreover, two key autophagy-associated proteins, ATG14 and ATG9A were downregulated (Fu et al., 2015). Another miRNA, bta-miR-375, decreased BMPR2, and ALK7 expression, resulted in attenuated proliferation ability and boosted the apoptosis rate of bovine cumulus cells (Chen et al., 2017). The MDBK cells is an important cell line for CPIV3 proliferation in our laboratory and it is efficient to produce CPIV3 in high titers. However, the relationship between the host cells miRNA and CPIV3 infection remains unclear. In the current study, in order to gain a better understanding of CPIV3 infection in MDBK cells at the miRNA level, high-throughput sequencing technology was used for small RNAs (sRNAs) in MDBK cells upon CPIV3 infection. We integrated analysis of miRNA profiles, and showed these miRNAs play an important role in regulating mRNA gene expression during CPIV3 infection. These data may provide a new method for the diagnostic and prevention of CPIV3-induced disease.

## Materials and methods

### Cell culture and virus strain

MDBK cells were maintained in Dulbecco′s modified Eagle medium (DMEM; SIGMA, USA) supplemented with 10% fetal bovine serum (FBS; HyClone, USA). The cultures were incubated at 37°C with 5% CO_2_. The CPIV3 JS2013 strain which was isolated in Jiangsu Province was used for virus infections.

### Virus infection and RNA isolation

MDBK cells grown to approximately 80–90% confluence, washed three times with phosphate-buffered saline (PBS), and infected with CPIV3 at a multiplicity of infection (MOI) of 10. After 1 h of adsorption, infected cells were maintained in fresh medium containing 2% FBS. Uninfected cells were used as a control. Each group was performed in triplicates. The CPIV3-infected and mock-infected group cells were harvested at 24 h post infection (hpi) and used for subsequent total RNA extraction.

### RNA isolation and sRNA sequencing

The total RNA from each group of MDBK cells was extracted using TRIzol UP reagent (Invitrogen, Carlsbad, USA) according to manufacturer′s instructions. The quantity and the concentration of total RNA in both samples were measured with an Agilent 2100 Bioanalyzer (Agilent Technoligies, Santa Clara, CA, USA) and a NanoDrop 2000 Spectrophotometer (Thermo Fisher Scientific, Waltham, MA, USA). sRNA sequencing was further performed in Huada Genomics Institute using the Illumina Genome Analyzer (Shenzhen, China). The steps were as follows: sRNA fragments between 18 and 30 nt were isolated from 15% denaturing polyacrylamide gel and ligated to a 5′ adaptor and 3′ adaptor. The ligated products were used as the templates for cDNA synthesis and amplified by PCR to enrich the libraries that were then used for cluster generation and sequencing.

### Analysis of sequencing data

The sRNA sequencing reads from the high-throughput sequencing were first cleaned by removing low-quality tags, adapter sequences and several types of contaminants. The clean reads from 18 to 30 nt were then mapped to the GenBank database (http://www.ncbi.nlm.nih.gov/), Rfam database (http://rfam.janelia.org/), and Repbase database (http://girinst.org/repbase/). After the sRNAs screened with the above-mentioned processes were excluded, the remaining short sRNAs were mapped to the miRNA precursors of the reference species (*B. taurus*), and the mature miRNAs were deposited in the miRBase 19.0 database (http://www.mirbase.org/). The unannotated short reads not mapped to miRBase 19.0 were subjected to novel candidate miRNAs prediction potentially using the Mfold RNA folding prediction web server (http://mfold.rna.albany.edu/).

### Differentially-expressed profile of miRNAs in resonse to CPIV3

In order to compare the differential miRNA expression in CPIV3-infected and Mock-infected MDBK cells, the numbers of raw tags in the two libraries were normalized using tags per million of the total miRNA reads (TPM). The miRNAs expressed at very low levels, a given low number (TPM < 10) to each miRNA value were excluded from the analysis. Changes in miRNA expression in CPIV3-infected vs. Mock-infected MDBK cells were considered significant when their *p* values were below 0.01. miRNAs with |log2 (fold changes)|>1 were designated as significantly upregulated or downregulated.

### Target prediction of miRNAs and fuctional analysis

The RNAhybrid (http://bibiserv.techfak.uni-bielefeld.de/rnahybrid/) and miRanda (http://www.microrna.org/) were used to predict the target genes. Gene ontology (GO) functional analysis against cell components, biological processes, and molecular functions were implemented (Ye et al., 2006). The KOBAS 2.0 annotation tool (http://www.genome.jp/kegg/pathway.html) was used to analyze a Kyoto Encyclopedia of Genes Genomes (KEGG) pathways.

### Analysis of the miRNAs and their mRNA targets by RT-qPCR

The expression of miRNAs were identified by high-throughput sequencing and then subjected to RT-qPCR assay. The RNA samples used for the high-throughput sequencing assays were also used for the RT-qPCR assay. Total RNA was first polyadenylated with polyA polymerase and then cDNA was synthesized with a poly(T) adapter primer (TIANGEN, Beijing, China). RT-qPCR was performed using an ABI Step One thermocycler (Applied Biosystems, CA, USA) with the miRcute miRNA qPCR SYBR Green Detection Kit (TIANGEN, Beijing, China) according to the manufacturer′s recommendations. The miRNA-specific forward primers using in this study are shown in Table 1. The 5S rRNA was used as an internal standard. Moreover, RT-qPCR was performed to examine the differentially expressed of mRNA targets. We used PrimeScript™ RT Master Mix (TaKaRa, Dalian, China) to synthesize first-strand cDNA. RT-qPCR was carried out using a SYBR Premix Ex Taq™ kit (TaKaRa, Dalian, China) following the manufacturer′s instructions. The specific primers for these mRNA targets are presented in Table 2. Three independent biological replicates were used for each gene. Relative expression level of each miRNA was calculated by the 2^−ΔΔct^ method.

**Table 1:** Primers used to confirm miRNA expression with RT-qPCR.

**Primer**	**Sequence (5′-3′)**
bta-miR-30d	TGTAAACATCCCCGACTGGAAGCT
bta-miR-574	TGAGTGTGTGTGTGTGAGTGTGTG
bta-miR-222	GAGCTACATCTGGCTACTGGGT
bta-miR-196a	GGCTAGGTAGTTTCATGTTGTTGGG
bta-miR-3613a	GCGGCTGTTGTACTTTTTTTTTTGTTC
bta-miR-1246	GGCAATGGATTTTTGGAGCAGG
bta-miR-2478	GCCGTATCCCACTTCTGACACCA
bta-miR-2904	GGGAGCCTCGGTTGGCCT
bta-miR-27a-5p	GGCTTCACAGTGGCTAAGTTCCG
novel-miR-51	TAATACTGCCTGGTAATGATGAC
novel-miR-99	TTGGGAAGCACAGACACTAGGACT
novel-miR-75	TCAAAGACTCGGACGTGACTGA
5S rRNA-F	GTCTACGGCCATACCACCCT

**Table 2:** Primers used to detect target genes expression with RT-qPCR.

**Primer**	**Sequence (5′-3′)**	**Product size (bp)**
Interleukin 2 (IL2)-F	CTTGTACCTCCGGTGTCGT	163
Interleukin 2 (IL2)-R	TGAAGAGTCAGGGAAGTTTGT	
Tumor protein p53 (TP53)-F	GAGCACTGCCTACCAACA	150
Tumor protein p53 (TP53)-R	CATCCAGAGCATCCTTCA	
Tumor necrosis factor (TNF)-F	TCGCTACATCACTGAACCT	82
Tumor necrosis factor (TNF)-R	GACACTTTATTTCTCGCCAC	
Nuclear factor kappaB (NF-κB)-F	TATCAAGCAGGTGGCAATCA	125
Nuclear factor kappaB (NF-κB)-R	TGGAGGAGGGTGGCAGA	
Natural killer cell triggering receptor (NKTR)-F	AAGAATTATGCTGGGAGT	175
Natural killer cell triggering receptor (NKTR)-R	AAGACTTAGTTGTGGGTTT	
Tumor protein p63 (TP63)-F	CAGCCAAGCATTCTCC	81
Tumor protein p63 (TP63)-R	AACGCAGCCTCTTATTT	

## Results

### Analysis of sRNA libraries from solexa sequencing

**Figure 1:**
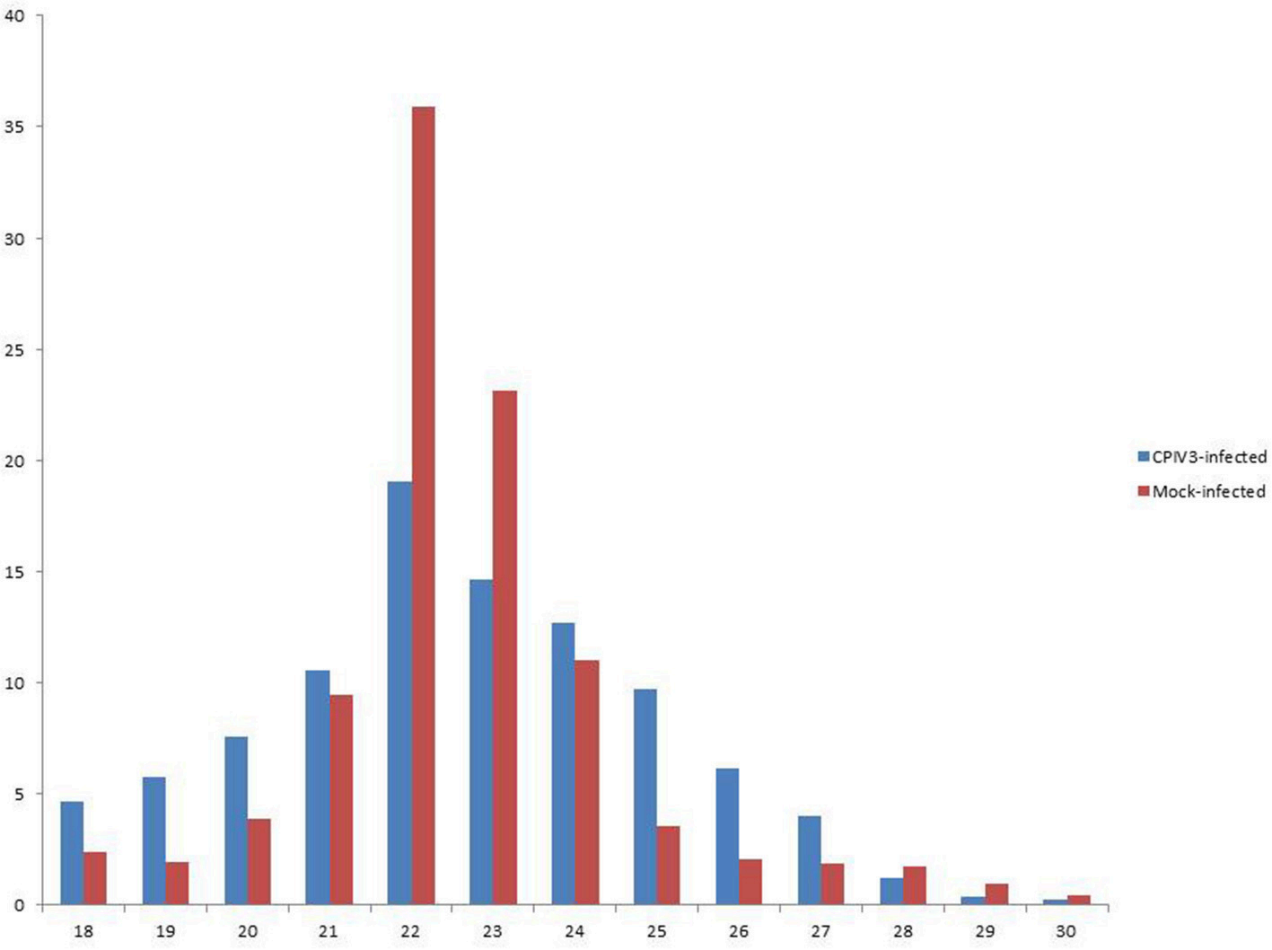
Length distribution of the clean reads of the sequences. The x-axis indicates the length of reads. The y-axis indicates the percentage of each length in the reads.

In order to reveal the effects of CPIV3 infection on MDBK cell miRNAs, two sRNA libraries from CPIV3-infected and Mock-infected MDBK cells were constructed and subjected to high-throughput sequencing technology. As shown in Table 3, the total numbers of raw reads collected from the infected and uninfected cells were 12,558,813 and 12,548,585, respectively. After removing low-quality tags, adapter sequences, and short reads smaller than 18 nt, 11,798,498 (infected) and 11,991,091 (uninfected) clean reads were identified. In addition, the final sRNA were annotated and classified as miRNA, rRNA, scRNA, snRNA, snoRNA, srpRNA, tRNA, exon-antisense, exon-sense, intron-antisense, intron-sense, and repeat (Table 3). The length distribution of the sRNA is presented in Figure 1. We found that most sRNA from both libraries were 21–24 nt in length, accounting for 10.55, 19.02, 14.61, 12.71 (infected), and 9.43, 35.9, 23.11, and 10.99% (uninfected) of sRNA.

**Table 3:** Distribution of sRNAs in CPIV3-infected and Mock-infected samples.

**Category**	**CPIV3-infected sample**				**Mock-infected sample**			
	**Unique**	**Percent**	**Total**	**Percent**	**Unique**	**Percent**	**Total**	**Percent**
Raw reads			12,558,813				12,548,585	
Clean reads	859,305		11,798,498		1,046,300		11,991,091	
Exon-antisense	928	0.11	1,378	0.01	2,486	0.24	3,441	0.029
Exon-sense	114,551	13.33	157,365	1.33	95,977	9.17	124,825	1.04
Intron-antisense	7,537	0.88	13,927	0.11	14,121	1.35	22,321	0.19
Intron-sense	63,353	7.37	123,735	1.05	243,410	23.26	402,426	3.36
miRNA	3,873	0.45	2,175,309	18.43	4,499	0.43	7,092,521	59.15
rRNA	98,164	11.42	5,141,485	43.58	62,742	6.00	990,300	8.26
repeat	26,432	3.07	52,282	0.44	84,920	8.11	132,151	1.10
scRNA	582	0.07	236,046	2.00	388	0.04	53,144	0.44
snRNA	14,389	1.67	180,738	1.53	6,058	0.58	25,812	0.22
snoRNA	15,682	1.83	93,468	0.79	8,856	0.85	91,274	0.76
srpRNA	1,449	0.17	38,943	0.33	524	0.05	1,704	0.01
tRNA	39,661	4.61	1,357,879	11.50	22,038	2.11	240,195	2.00
unannotated	472,668	55.01	2,225,943	18.87	500,281	47.81	2,810,977	23.44

### Identification of known miRNAs in MDBK cells

**Figure 2:**
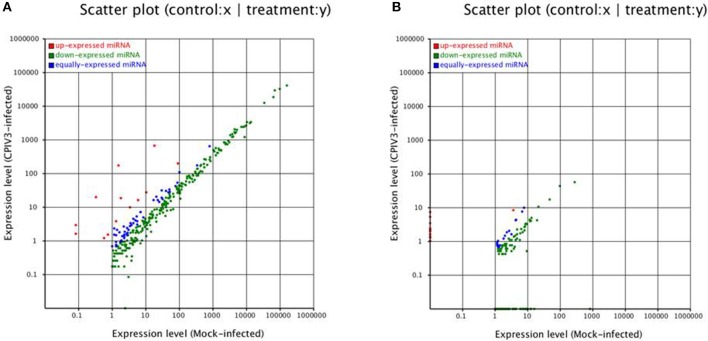
Differential expression levels of known miRNAs **(A)** and novel miRNAs **(B)** in CPIV3-infected and mock-infected groups. The x and y axes show the differential expression levels of miRNAs of the two groups. The red points represent up-expressed miRNAs with a ratio >2, the blue points represent equally- expressed miRNAs with a ratio ≥1/2 and ≤ 2, and the green points represent down- expressed miRNAs with a ratio <1/2.

**Figure 3:**
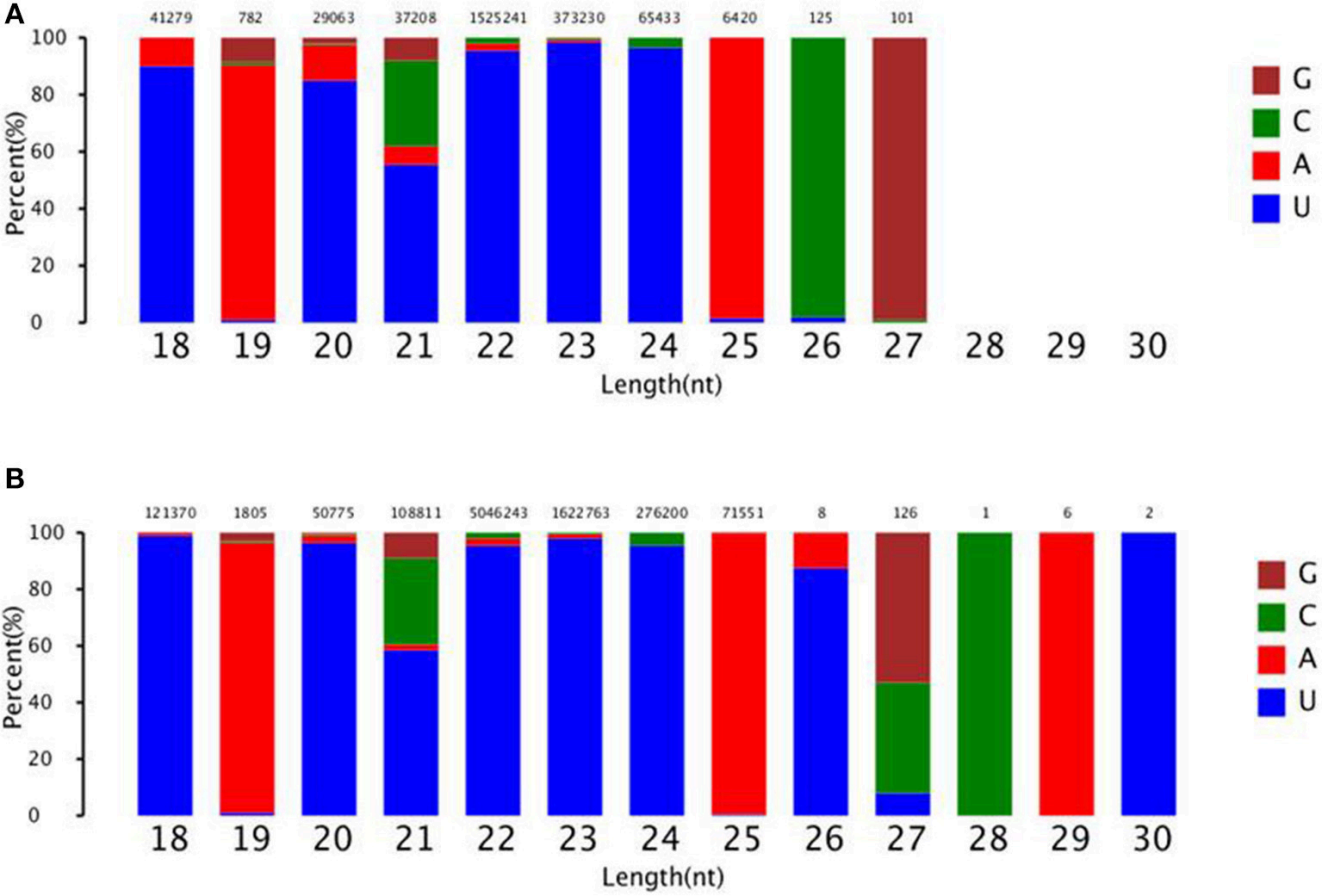
The ranged in size and base bias at the first position of miRNA identified in CPIV3-infected and mock-infected cells. **(A)** CPIV3-infected cells **(B)** mock-infected cells. The x-axis indicates miRNAs lengths from 18 to 30 nt. The y-axis indicates the percentage of the base bias of miRNAs at the first position of each length.

To identify known miRNAs change in MDBK cells infected with CPIV3, The sRNAs sequences were mapped to the known mature miRNAs and their precursors in miRBase 20.0 database to obtain the miRNA count as well as the base bias at the first position. Approximately 3,873 unique sequences (2,175,309 reads) in the infected library and 4,499 unique sequences (7,092,521 reads) in the uninfected library were annotated as miRNA candidates (Table 2). A total of 363 and 321 known miRNA genes were identified in the CPIV3-infected and Mock-infected MDBK cells libraries, respectively. Different expression patterns of miRNAs between the two groups were showed in Heat map and Scatter Plot (Supplementary Data Sheet 1). Using a *P* < 0.01 and a |log2 (CPIV3-infected/Mock-infected in expression) |>1 as the cut-off values, a total number of 249 differentially expressed known miRNAs were identified in the two groups, 9 were upregulated and 240 were downregulated (Supplementary Data Sheet 2 and Figure 2A). It is notable that most of the miRNAs were downregulated after CPIV3 infection. Additionally, the majority of miRNAs had lengths of 22 or 23 nt, and the first nucleotide bias in identified miRNAs showed a strong preference for ′U′ at the 5′-end (Figure 3), which is consistent with previous studies (Zhang et al., 2016).

The miRNA conservations were analyzed using the available genome assemblies of other mammal, and the conservation of 255 known miRNAs across four other species (Capra hircus, Ovis aries, Equus caballus and Sus scrofa) is shown in Supplementary Data Sheet 3. Of these 255 miRNAs, 85 were not found in Capra hircus and Ovis aries; 31 were not found in all four species, whereas, 29 miRNAs were found in all these four species.

### Identification of novel miRNAs in MDBK cells

The remaining unannotated sRNAs against the *B. taurus* genome were used to predict novel miRNAs in MDBK cells. A number of unannotated sRNAs, 2,225,943 and 2,810,977 were present in the CPIV3-infected and Mock-infected groups, respectively, and these sRNAs were used to predict novel miRNA candidates. A total of 234 and 447 novel miRNAs were predicted in the CPIV3-infected and Mock-infected MDBK cells libraries using miReap software are shown in Supplementary Data Sheets 4, 5. Dicer cleavage site, minimum free energy, frequency of reads and typical secondary structures of the characteristic stem loop hairpins were showed in the two files. Moreover, we revealed different expression patterns of miRNAs between the two groups in heat map and Scatter Plot (Supplementary Data Sheet 6). Based on the cut-off values of the differential expression analysis demonstrated previously, 152 novel candidate miRNAs were identified in the two groups, 16 miRNAs were upregulated and 136 of which were downregulated (*P* < 0.01) (Supplementary Data Sheet 7 and Figure 2B).

### Target genes prediction for differentially expressed miRNAs

In order to investigate the biological functions of the differentially expressed miRNAs, two independent algorithms, miRanda and RNAhybrid were used to predict the mRNA targets. A total of 95,389 and 92,412 genes for 249 known miRNAs and 152 novel miRNAs, respectively, were predicted as potential miRNA targets (Supplementary Data Sheets 8, 9). GO analysis of the predicted target genes revealed that 22,981 and 22,572 target genes were annotated successfully for the 249 known miRNAs and 152 novel miRNAs, respectively, and they were involved in biological process, cellular component and molecular function (Supplementary Data Sheets 10–12). In order to explore the roles of miRNAs might play in regulatory networks, KEGG Orthology Based Annotation System (KOBAS) analysis was performed. The results showed that most of the abundant KEGG terms were involved in biological processes such as pathways in cancer (ko05200), focal adhesion (ko04510), endocytosis (ko04144), MAPK signaling pathway (ko04010), p53 signaling pathway (ko04115), Fc gamma R-mediated phagocytosis (ko04666), B cell receptor signaling pathway (ko04662), synaptic vesicle cycle (ko04721), chronic myeloid leukemia (ko05220), and synaptic vesicle cycle (ko04721) (Supplementary Data Sheet 13).

### Validation of miRNAs and mRNA by RT-qPCR

**Figure 4:**
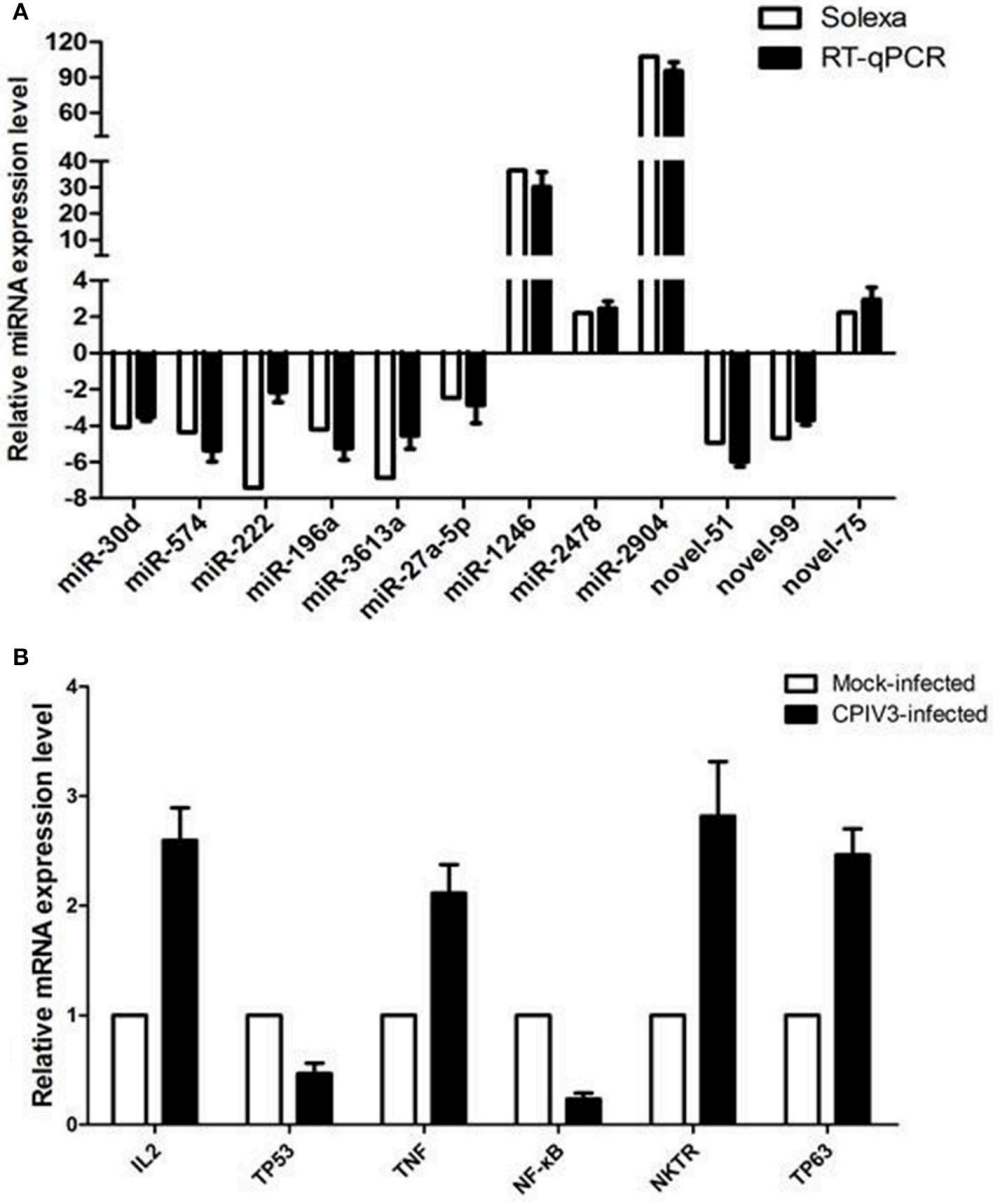
Validation of miRNA **(A)** and mRNA targets **(B)** expression by qRT-PCR. The fold change of expression of 12 miRNAs and their mRNA targets in CPIV3 infected vs. CPIV3 uninfected MDBK cells was calculated using the 2-ΔΔct method and represented as the n-fold change.

RT-qPCR assay was performed to further investigate the differentially expressed miRNAs from sequencing data. Nine known miRNAs and three novel candidate miRNAs were selected for validation. The results confirmed the consistent expression profiles with the sRNAs sequencing data. Results confirmed the down-regulation of 8 miRNAs (novel-miR-51, novel-miR-99, miR-30d, miR-574, miR-222, miR-196a, miR-3613a, and miR-27a-5p) and the up-regulation of 4 miRNAs (novel-miR-75, miR-1246, miR-2478, and miR-2904) in infected MDBK cells compared with the uninfected (Figure 4A).

**Figure 5:**
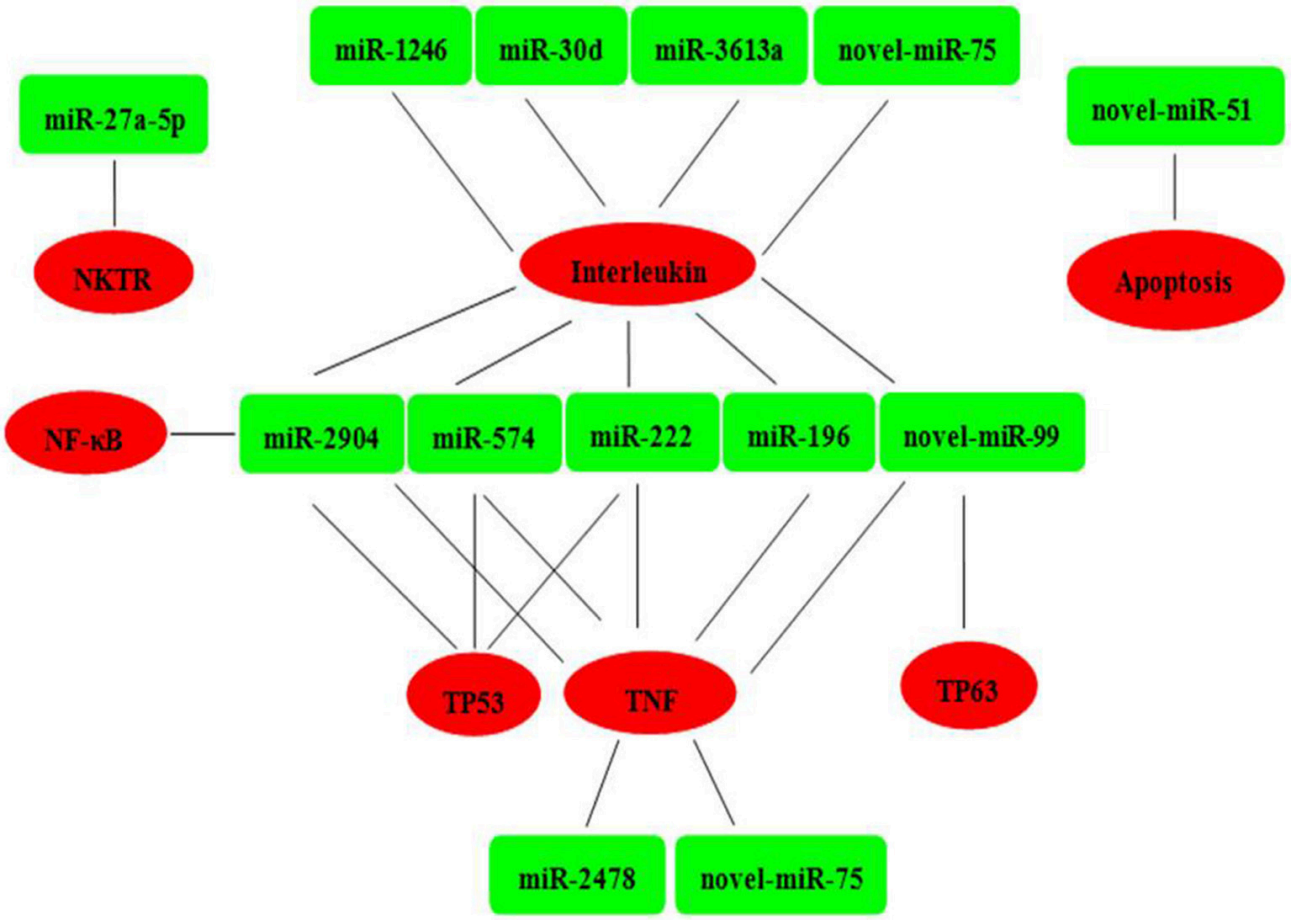
Relationships between miRNAs and inversely correlated immune target genes. Green indicates miRNAs; red indicates immune target genes.

Six mRNAs that were targeted by 12 differentially expressed miRNAs were selected to perform RT-qPCR assay. As a results, 4 targets (IL2, TNF, NKTR, and TP63) were upregulated, 2 targets (TP53 and NF-kappa B) were downregulated (Figure 4B). The network of interaction between miRNAs and their targets was shown in Figure 5.

## Discussion

In recent years, researchers used high-throughput sequencing technology to reveal the expression profiles of miRNAs in animals, plants, and viruses. Integration analysis of miRNAs and mRNAs in virus-infected sample helped to demonstrate miRNA regulatory mechanisms (Wang et al., 2012). Nevertheless, it is unknown whether celluar miRNAs effected CPIV3 replication by modulating the host immune responses and targeting viruses. To this end, high-throughput sequencing approache was subjected to identify cellular miRNAs involved in the response to CPIV3 in MDBK cells. A total of 11,798,498 and 11,991,091 clean reads were obtained from CPIV3-infected and uninfected samples, respectively. Subsequently, 249 know miRNAs and 152 novel miRNAs that were differentially expressed in CPIV3-infected and uninfected cells were identified successfully. It was possible that these miRNAs may involved in the interaction between MDBK cells and CPIV3.

RNAi is a fundamental innate immune pathway in eukaryotic cells as the first line against invading pathogens, and it has been broadly studied recently (Akinc et al., 2008; Whitehead et al., 2009). The siRNA-based anti-viral therapies is obvious shortcoming, for requiring complete sequence complementarity to the target RNA genes. Nevertheless, miRNAs do not require complete complementarity to their target RNA molecules compare with siRNAs, and regulate the gene expression for a variety of biological processes (Lam et al., 2015). Previous studies showed that bovine viral diarrhea virus (BVDV) and bovine herpesvirus 1 (BoHV1) infections of MDBK cells can influence the expression of cellular miRNAs (Glazov et al., 2009; Fu et al., 2015). In this study, the majority of clean reads in CPIV3-infected and uninfected-cells had lengths of 21–24 nt, and the 22 nt RNAs were the most abundant, accounting for 19.02 and 35.9% of sRNA in the infected and uninfected libraries, respectively. These results were consistend with the typical size of miRNA from Dicer-derived products, and suggested that the libraries were highly enriched in miRNA sequences.

In bovines, the altered miRNAs expression profiles were occured during viral infections, and led to positively or negative regulate host immune (Vegh et al., 2013; Stenfeldt et al., 2017). More recently, Stenfeldt et al. revealed that bta-miR-17–5p and bta-miR-31 were highest expressed during acute, persistent infection, respectively, whereas, bta-miR-1281 was significantly downregulated while both acute and persistent infection of FMDV (Stenfeldt et al., 2017). Another study elucidated that bta-miR-146a significantly inhibited the expression levels of TRAF6 and NF-kappa B, and suppressed bovine inflammation and innate immune responses through down-regulation of the TLR4/TRAF6/NF-kappa B pathway (Wang X. P. et al., 2017). To date, researchers have demonstrated that several *Bos taurus* miRNAs have an antiviral activity against pathogens. A recent study showed that the expression of bta-miR-29b was upregulated in response to BVDV infection in MDBK cells, and bta-miR-29b overexpression led to the attenuation of BVDV infection-related autophagy by directly downregulating the autophagy-associated proteins, ATG14 and ATG9A (Fu et al., 2015). Further study demonstrated that the bta-miR-29b decreased the levels of BVDV envelope glycoprotein E1 mRNA to suppressed viral replication, and attenuated apoptosis by directly regulating intracellular levels of caspase-7 and nuclear apoptosis-inducing factor 1(NAIF1) (Fu et al., 2014). Whereas, bta-miR-193a was reported to promoted apoptosis and inhibited BVDV replication by target the 3′-untranslated region (UTR) of B-cell lymphoma-2-associated X protein (BAX) mRNA. In the current study, we found that bta-miR-29b and bta-miR-193a were down regulated upon CPIV3 infection. These results indicate the possibility that bta-miR-29b and bta-miR-193a likely participate in host-virus interaction in MDBK cells. Previous reports have shown that host miR-23 and miR-26a inhibited porcine reproductive and respiratory syndrome virus (PRRSV) replication *in vitro* strongly by activating the type I interferon (IFN) signaling pathway and promoting the expression of IFN-stimulated genes (Zhang et al., 2014; Jia et al., 2015; Li et al., 2015). In addition, IFN-gamma inducible protein (IP-10) was negatively correlated with miR-21. Increased expression of miR-21 was shown to promote pseudorabies virus (PRV) replication by targeting IP-10 (Huang et al., 2014). In our study, miR-23, miR-26a, and miR-21 were downregulated following CPIV3 infection, respectively. These results indicate the possibility that these miRNAs may also be involved in interactions between *Bos taurus* hosts and CPIV3.

The target genes of the 249 miRNAs were further predicted, and we selected miRNA-mRNA correlation pairs for RT-qPCR assay. The expression profiles of 12 miRNAs were consistent with the sRNA sequencing results. The 12 differentially expressed miRNAs was negatively correlated with the expression of the mRNA targets, which indicates an important regulatory role of the miRNAs. In general, most miRNAs were inversely correlated with several mRNA target and some mRNAs were targeted by some of miRNAs (Krutzfeldt et al., 2006). GO analysis showed those mRNA targets inversely correlated with miRNAs were involved in biological regulation, immune system processes, responses to stimuli, and other cellular processes. The KEGG pathway analysis further demonstrated that these target genes were mainly involved in significant cellular pathways, including Toll-like receptor signaling pathway, Jak-STAT signaling pathway, MAPK signaling pathway, p53 signaling pathway, focal adhesion, pathways in cancer, NF-kappa B signaling pathway, apoptosis, regulation of autophagy. The MAPK, p53, Jak-STAT, and Toll-like receptor signaling pathway are involved in apoptosis and innate immunity (Takaoka et al., 2003; Sun et al., 2012), which may be related to CPIV3 replication in MDBK cells. Previous study showed that sendai virus (Bitzer et al., 1999), canine distemper virus (CDV) (Guo and Lu, 2000), newcastle disease virus (NDV) (Ravindra et al., 2008), and peste des petits ruminants virus (PPRV) (Mondal et al., 2001), which belong to the *Paramyxiviridae* family were able to induce apoptosis during infections of various cell cultures. Apoptosis is a process of programmed cell death process that lead to host cells changes and death in response to infection in order to limit viral replication (Labbe and Saleh, 2008). Whereas, viruses may benefit from stimulating apoptosis, the breakdown of infected cells in the host, thereby favoring viral dissemination (Galluzzi et al., 2008). Moreover, innate immunity is the first line of defense against viral infection, and IFN are potent immune responsive cytokines against invading viruses. Therefore, it will be very interesting to confirm induction of apoptosis and IFN-related signaling pathway in MDBK cells in response to CPIV3 infection in the future.

## Conclusion

In summary, using high-throughput sequencing technology, the expression patterns in MDBK cell line followed by CPIV3 infection were evaluated. A total of 249 known differentially expressed miRNAs and 152 novel candidate miRNAs were identified from the two samples. Subsequently, 12 of miRNAs and their target genes were confirmed by RT-qPCR. Target prediction and functional analysis of these miRNAs suggested that they may play an important role in regulating CPIV3 replication and the host immune response. This is the first report to integrate miRNA expression data in infected MDBK cells. We believe that these data will contribute to further study the pathogenic mechanism of CPIV3.

## Author contributions

JL took part in all the experiments and wrote the manuscript. LM, WL, and JJ helped to design the whole project and draft the manuscript. FH, CZ, LY, WZ, and ML conducted RNA isolation and sample processing for sequencing. XZ and XJ conducted data analysis. All authors read and approved the final manuscript.

### Conflict of interest statement

The authors declare that the research was conducted in the absence of any commercial or financial relationships that could be construed as a potential conflict of interest.

## Supplementary material

Supplementary Data Sheet 1 Heat map of known miRNAs.

Supplementary Data Sheet 2 Differentially expressed known miRNAs in the two groups.

Supplementary Data Sheet 3 Analyzed of miRNA conservations using other mammal.

Supplementary Data Sheet 4 Predicted of novel miRNAs in CPIV3-infected group cells.

Supplementary Data Sheet 5 Predicted of novel miRNAs in mock-infected group cells.

Supplementary Data Sheet 6 Heat map of novel miRNAs.

Supplementary Data Sheet 7 Novel miRNAs were differentially expressed in the two groups.

Supplementary Data Sheet 8 Predicted of the mRNA targets of the differentially expressed known miRNAs.

Supplementary Data Sheet 9 Predicted of the mRNA targets of the differentially expressed novel miRNAs.

Supplementary Data Sheet 10 GO annotation of the predicted target genes from differentially expressed known and novel miRNAs.

Supplementary Data Sheet 11 GO annotation of the predicted target genes from differentially expressed known miRNAs.

Supplementary Data Sheet 12 GO annotation of the predicted target genes from differentially expressed novel miRNAs.

Supplementary Data Sheet 13 The regulatory networks of miRNAs.
