# Supplementary figures and images for: Analysis of microRNAs Expression Profiles in Madin-Darby Bovine Kidney Cells Infected With Caprine Parainfluenza Virus Type 3
Source: Front Cell Infect Microbiol. 2018 Mar 29;8:93. doi: 10.3389/fcimb.2018.00093 (PMC5885596; doi:10.3389/fcimb.2018.00093)

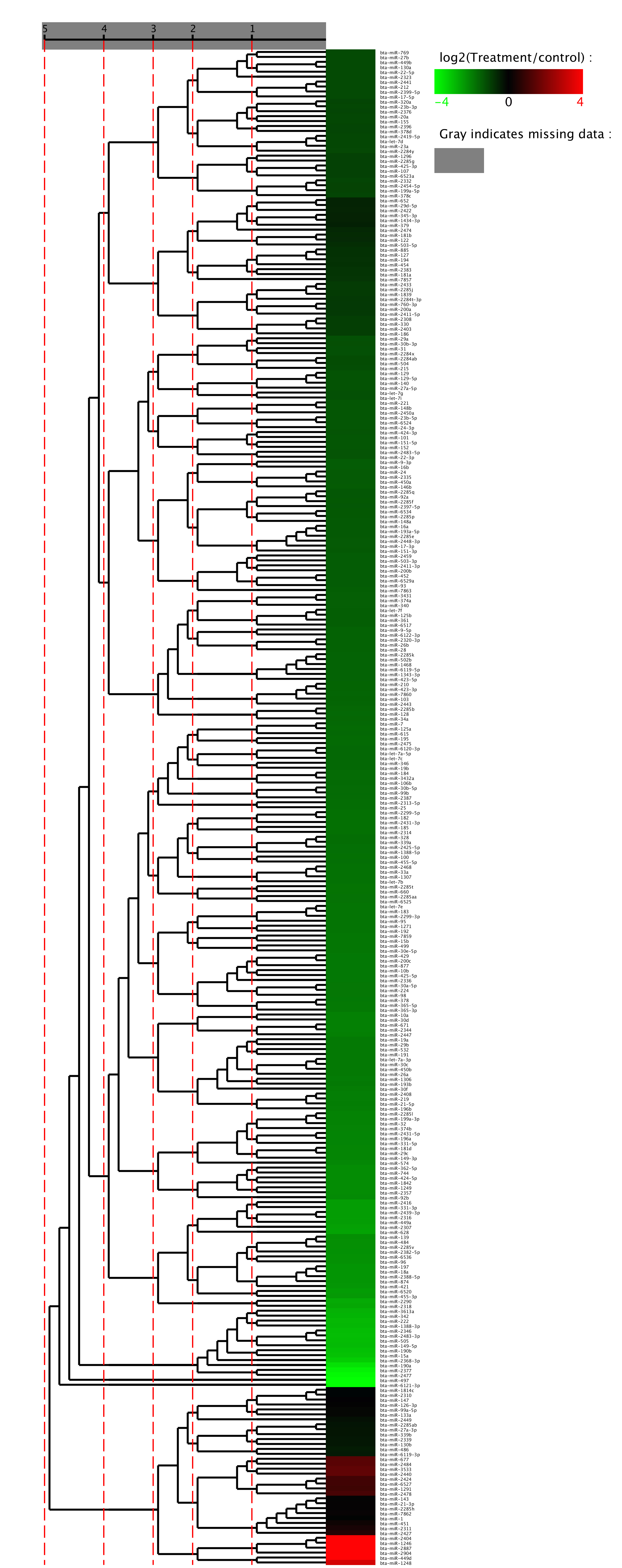

Supplement: Supplementary Data Sheet 1 — Heat map of known miRNAs. [file DataSheet1.zip › image 1.jpeg]

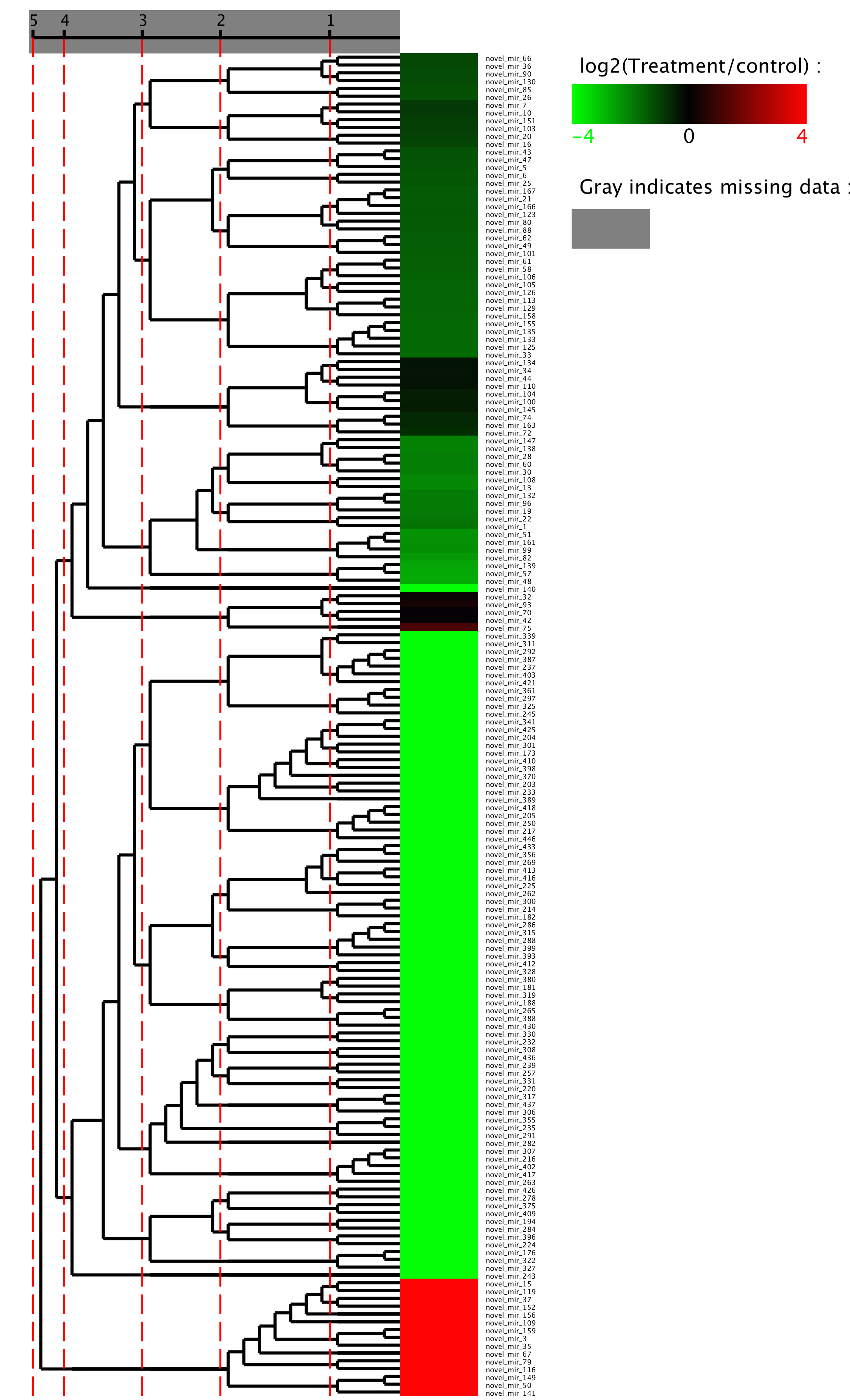

Supplement: Supplementary Data Sheet 6 — Heat map of novel miRNAs. [file DataSheet6.zip › Image 6.JPEG]

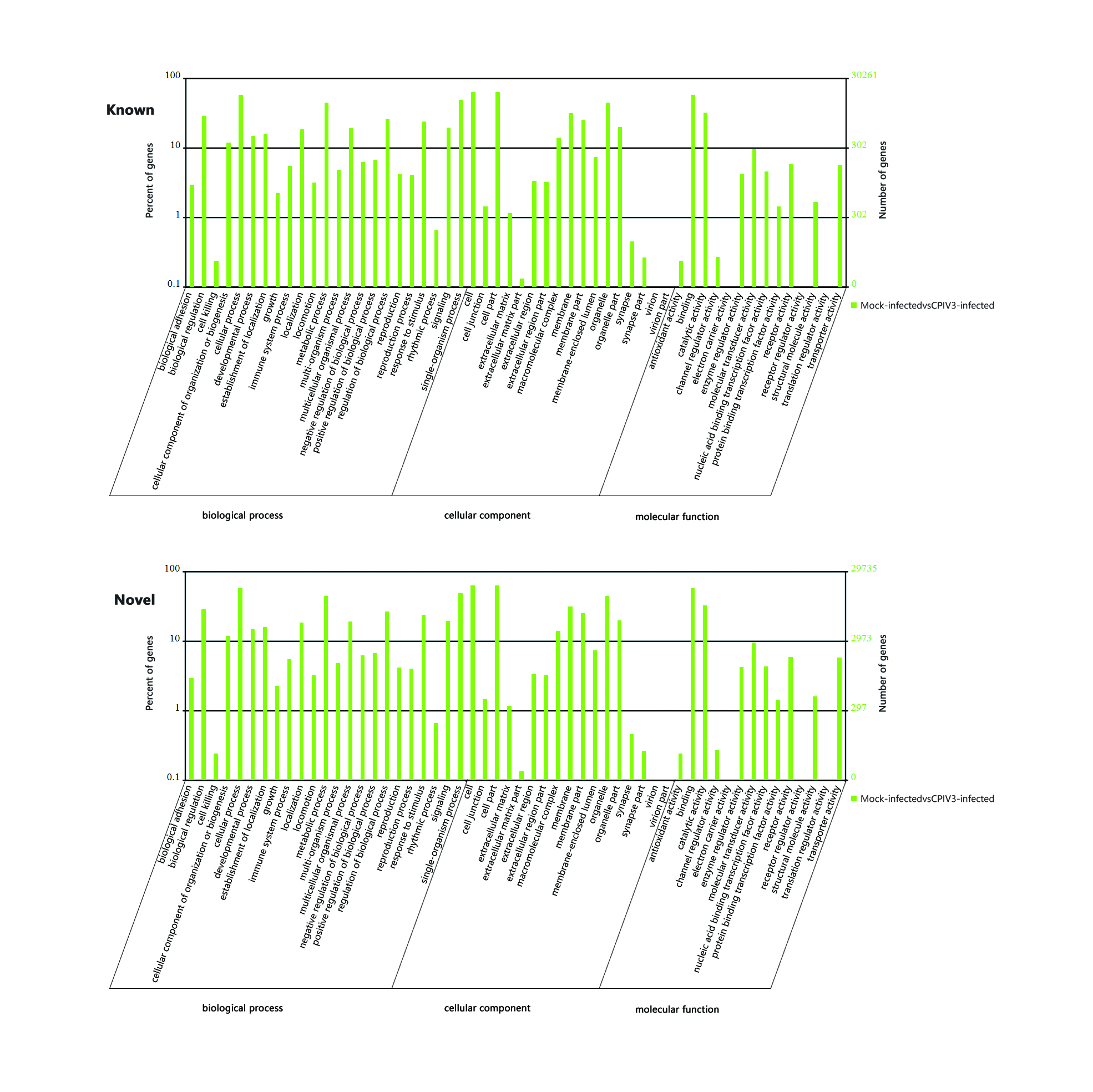

Supplement: Supplementary Data Sheet 10 — GO annotation of the predicted target genes from differentially expressed known and novel miRNAs. [file DataSheet10.zip › Image 10.JPEG]
